# Supplementary material for: Genetics of Ataxias in Indian Population: A Collative Insight from a Common Genetic Screening Tool
Source: Adv Genet (Hoboken). 2022 Mar 10;3(2):2100078. doi: 10.1002/ggn2.202100078 (PMC9744545; doi:10.1002/ggn2.202100078)
Supplement: Supplementary file 1 — Supporting information [file GGN2-3-2100078-s001.pdf]

Supporting Information

Genetics of ataxias in Indian population: A collative insight from common a genetic screening tool

Pooja Sharma [MSc,1,2], *Akhilesh Kumar Sonakar* [PhD, 3], *Nishu Tyagi*[MSc,1,2], *Varun Suroliya* [PhD, 3], *Manish Kumar* [MSc,1,2], *Rintu Kutum*[Phd,1],*Vivekananda A* [MSc,1,2], *Sakshi Ambawat*[MSc,1], *Uzma Shamim* [PhD, 1], *Avni Anand*[1], *Ishtaq Ahmad*[PhD,3], *Sunil Shakya*[PhD,3],*BharathramUppiliBtech*[,1,2], *Aradhana Mathur*[PhD,1], *Shaista Parveen*[MSc,1], *Shweta Jain*[MSc,1], *Jyotsna Singh*[MSc1,3], *Malika Seth*[M.Tech,1], *Sana Zahra*[MSc,1,2], *Aditi Joshi*[MSc,1], *Divya Goel*[M.S.Pharm,1], *Shweta Sahni*[MSc,1], *Asangla Kamai*[MSc,1,2], *Saruchi Wadhwa*[MSc,1,2], *Aparna Murali*[Btech1], *Sheeba Saifi*[MSc1], *Debashish Chowdhury* [DM,4], *Sanjay Pandey* [DM,4], *KS Anand* [DM,5], *R.Lakshmi Narasimhan* [DM,10], *Sanghamitra Laskar* [DM,6], *Suman Kushwaha* [DM,7], *Mukesh Kumar* [DM,8], *C.V Shaji*[DM,9], *MV Padma Srivastava*[DM,3], *Achal K Srivastava* [DM, 3] *Mohammed Faruq* [MBBS, PhD 1,2*] *and GOMED-Ataxia study group*[11]

SCA 1		SCA 2		SCA3		SCA6		SCA7		SCA12		SCA17		FRDA	
**CLINICAL DIAGNOSTICS**	**Number of CD**	**CLINICAL DIAGNOSTICS**	**Number of CD**	**CLINICAL DIAGNOSTICS**	**Number of CD**	**CLINICAL DIAGNOSTICS**	**Number of CD**	**CLINICAL DIAGNOSTICS**	**Number of CD**	**CLINICAL DIAGNOSTICS**	**Number of CD**	**CLINICAL DIAGNOSTICS**	**Number of CD**	**CLINICAL DIAGNOSTICS**	**Number of CD**
ADCA	85	SCA2	165	ADCA	29	ADCA	4	ADCA	11	SCA12	250	ADCA	1	FRDA	138
SCA1	55	ADCA	106	SCA3	20	SCA6/12	1	SCA7	8	ADCA	63	ARCA/FRDA	1	ARCA	11
SCA2	21	SCA12	15	SCA1	10			SCA1	2	OTHERS	4	Parkinsonism	1	ADCA	11
SCA3	7	SCA1	12	SCA2	6			ADCA/MSA	1	SCA1	3	OTHERS	1	SCA1	3
ADCA/ARCA	4	FRDA	7	ADCA/MSA	1			OTHERS	1	SCA2	3	SCA+	1	SCA2	2
SCA12	4	ARCA	6	ARCA	1			SCA+	1	SCA+	2			SCA3	1
FRDA	2	ADCA/MSA	4	ARCA/AT	1			SCA12/ET	1	SCA17	2			SCA12	1
MSA	2	ADCA/FRDA	3	FRDA	1			SCA2	1	ADCA/MSA	1				
SCA+	2	SCA3	3	SCA+	1					ARCA/FRDA	1				
ARCA	1	ARCA/FRDA	2	Tremor	1					ET/SCA2	1				
SCA17, SCA2	1	MSA	2							FRDA	1				
SCA2/SCA3	1	OTHERS	2							FRDA/SCA1	1				
sca6,sca7	1	ADCA/ARCA	1							MSA	1				
SCA7, SCA4	1	AT	1							SCA/ARCA	1				
		ET	1							SCA7	1				
		HD	1												
		MND	1												
		SCA7	1												

Table-S1: Table illustrating related clinical diagnosis of SCA-subtypes referred (GOMED-Ataxia Study Group from 2015 to 2021)

Supporting material 2: GOMED-Ataxia Study Group:

1. Ankit Goyal[DM], Garima shukla[DM], Madhulika Kabra[DM], Radhakrishna[DM], Roopa Rajan[DM], Deepika[DM], Amrita Gotur[DM], Biswamohan Mishra[DM], Rahul Singhal[DM], Rahul Oinam[DM], Vibhor Upadhaya[DM], Prashant Bhatele[DM], Deepak Yadav[DM], Rahul Chawala[DM] (Neurology Department, Neuroscience Centre, All India Institute of Medical Sciences, New Delhi, India)
2. Debashish Chowdhury [DM],Sanjay Pandey [DM], Ashish kumar Duggal [DM], Swapan gupta[DM], GA Khwaja[DM], Neera Chaudhry[DM], Dilip Nagarwal[DM], Apoorva Tomar[DM], Sonali Bhattad[DM], Anumeha Mishra[DM], Vaibhav[DM], Meera Chaudhary[DM], Sunil. K. pollipalli[DM], Ankit Shah[DM], Avishkar[DM], Rajena[DM], Ralaskar[DM], Tanvi Shukla[DM], Ankit Muna[DM] , Pradeep Bijaynath Tiwari[DM] , Ashwin K Panda, Arun Kaul, Luv Bansal, Sujata Roshan, Sunil Agrawal (Department of Neurology, Govind Ballabh Pant Institute of Postgraduate Medical Education and Research, JLN Marg, New Delhi)
3. KS Anand [DM], Rahul Jain[DM], Saurabh Gupta, Amit Gupta, Mansi Shah, Brijlal, Bhuwan Sharma, Sruthi, Rashmi Singh, Vinod Kumar Jain, Arun k Agrawal, Rahul Mahajan, Irteqa Ali, Harish Gupta (Ram Manohar Lohia Hospital, New Delhi)
4. A.Nalini[MD], Pramod Pal, Rashmi, Srijithesh (National Institute Of Mental Health And Neuro Sciences, Banglore)
5. R.Lakshmi Narasimhan[DM], Deepa, Balasubramanian, Nagaraj A R (Institute of Neurology, Madras Medical College, Chennai)
6. Sanghamitra Laskar [DM] (VMMC and Safdarjung Hospital, New Delhi)
7. Samhita Panda[DM] (Department of Neurology AIIMS, Jodhpur)
8. Deepika Joshi [DM] (Banaras Hindu University, Varanasi)
9. Sweta single[DM] (St Stephan's Hospital)
10. Naresh Tayade [MBBS] (LifeCare Child Care Hospital, Amrawati, Maharastra)
11. Seema Kapoor[DM] (Maulana Azad Medical College, New Delhi)
12. Abhishek Pathak[MD], Rameshwarnath Chaurasia, Royana Singh (Banaras Hindu University, Varanasi)
13. Suman Kushwaha[DM], Padma, Ashutosh Gupta, Sheetal goyal, Monika Sachan, Balakrishan, Shaikh Azeez, Arpit Kumar Jain, Nitish Seth, Atul Yadav, Vivek Barvn, Neha Aggarwal, Anthony, Arvind G, Alin, Aravind Gunasekaran (Institute of Human Behaviour and Allied Sciences, New Delhi)
14. Vinod Puri [DM], Mukesh Kumar, J.D.Mukherjee (Max Super Speciality Hospital, Saket, New Delhi)
15. Soumya Sundaram [DM], Poornima Narayanan Nambiar, Sapna, Asha Kishore, M.D Nair, Syam K, Vyshaka K V , Ashish AS (Sree Chitra Tirunal Institute for Medical Sciences and Technology, Trivandrum)
16. C.V Shaji[DM], PK Muhammad, Kabeer K A (Govt. T.D. Medical College, Alappuzha)
17. Vivek jain[MD] (Santokba Durlabhji Memorial Hospital and Medical Research institute, Jaipur, India)
18. Ram kumar S.[DM], Rajeswari Aghoram, Rajesh Reddy P., Sourabh Jain, Aian Noronha, Jithin Mathew, Sunil K, Narayan, Balachanan, Indira priya, Chanashekara MN (Jawaharlal Institute Of Postgraduate Medical Education And Research, Puducherry, India)

19. Srikumar (Govt.Medical College, Trivan , Pattom,Thiruvananthapuram)
20. Sangeeta Rawat (King Edward Memorial Hospital, Mumbai)
21. K Shanmughasundaram [MD] (Apollo Clinic, Velachery, Chennai)
22. R M Bhoopathy (Apollo Hospitals Tondiarpet, Chennai)
23. Arun K Valsan (Armed Force Medical College, Pune)
24. M. A Joy (Ashwini Hospital, Thrissur, Kerala)
25. Pradeep Reddy (Chennai Medical College, Trichy)
26. Gaurav Kumar Mittal Delhi (Heart and Lung Institute, New Delhi)
27. Ruby chopra (Delhi heart and multispeciality hospital, Bathinda)
28. Manish Parakh (Dr S N Medical College, Jodhpur)
29. Jayesh Sheth (Foundation for Research in Genetics and endocrinology , The Institute of Human Genetics, Ahemdabad Moyinul Haq EMS Memorial Hospital, Perinthalmanna, Kerala)
30. G.Sarala [DM] (I J Multi specialities,Purasawakkam, Chennai)
31. Ramdas Solanke (Indira Gandhi Institute of Medical Science, Sheikhpura, Patna)
32. Manisha Goyal (J k Lone Hospital, Jaipur)
33. Shruti Jain (Jaipur Golden Hospital, New Delhi)
34. Suvasini (Kalawati Hospital, New Delhi)
35. Anju Shukla, Girisha KM (Kasturba Medical College, Manipal)
36. Praful Singh[MS, PhD], Arvind Kumar, RK Dhamija, Dhanjai, Devyani (Lady Hardings Medical College, New Delhi)
37. S. Anuradha (Lok Nayak Hospital, New Delhi)
38. Karuna (Maharaja Yeshwantrao Hospital, Indore)
39. Apoorva Pauranik (Mahatama Gandhi Memorial Medical College, Indore)
40. Amit Vyas [DM] (Meera Neuro Clinic, Indore)
41. J N Goswami (RR Hospital, New Delhi)
42. PS Rathi (Rathi's Mind Centre, Vijay Nagar, Indore)
43. Husna Shamim, Meena Lamjiwar, Himank Goel, Buddhi Prakash, Anirudh Rao Deshmukh, Chandan, Ananya Sengupta (Safdarjung Hospital, New Delhi)
44. S. S Bedi (Sharanjit Hospital, Jhalandar)
45. Chana Shekhar, Rahul (Sir Ganga Ram Hospital, New Delhi)
46. Soham Desai [DM] (Shree Krishna Hospital, Karamsad, Gujrat)
47. raveen Paniker (Sree Gokulam Medical College, Trivandrum)
48. Rishu Garg[DM] , Hashash Singh Ishar[DM], Amandeep Singh, Dinesh chouksey, Rohit Prabhas (Sri Aurobindo Institute of Medical Sciences , Indore)
49. Ashish Sushvirkar [DM] (Sri Manakula Vinayagar Medical College and Hospital, Puducherry)
50. Marian Jude Vijay[DM] (Tamil Nadu Govt. Multi Superspeciality Hospital)
51. Tapaswi Puwar[MD] (The Indian Institute of Public Health Gandhinagar, GandhiNagar)
52. K. Shantaraman[DM] (Tirunelveli Medical College, Tirunelveli)
53. Kishore KV[DM] (Vydehi Institute of medical sciences and research centre, Whitefield Bangalore)
